# P53 abnormalities and outcomes in colorectal cancer: a systematic review

**DOI:** 10.1038/sj.bjc.6602589

**Published:** 2005-04-26

**Authors:** F M Smith, R B Stephens, M J Kennedy, J V Reynolds

**Affiliations:** 1 University Department of Surgery and The Department of Clinical and Molecular Oncology, St James's Hospital, Dublin 8, Ireland

**Sir**,

We would like to make comment on the above article, which as its main conclusion found that abnormal p53 was associated with failure of response to radiotherapy in patients with rectal cancer. Our department has recently conducted a similar analysis focusing purely on rectal cancer undergoing neoadjuvant treatment and the conclusions that we drew were somewhat different.

In total, we sourced 22 studies that assessed p53 status in this setting. Of these, 18 used immunohistochemistry (IHC) and only four (18%) showed that it could predict response. A further six studies assessed the *p53* gene directly, four using single-strand conformational polymorphism analysis (ssCP) (Sakakura *et al*, 1998; Elsaleh *et al*, 2000; Rau *et al*, 2003; Saw *et al*, 2003) and two using direct *p53* gene sequencing (DGS) (Kandioler *et al*, 2002; Rebischung *et al*, 2002). No study (0%) where ssCP was used could predict response. While both the studies where DGS was used showed that tumours manifesting mutations in p53 were less likely to respond, we feel that these results should be interpreted with caution for several reasons.

Firstly, patients in both DGS studies underwent short-course radiotherapy, whereas there is an increasing use of long-course neoadjuvant radiochemotherapy (RCT) as standard of care in many institutions (Sauer *et al*, 2004). Also, the pathological end point used in both studies was a decrease in T stage determined by comparison of pretreatment transrectal ultrasound (TRUS) and postoperative pathological staging, which may be unreliable. For example, a recently published study of 1184 patients undergoing pretreatment staging by TRUS found that it had an overall staging accuracy of 69% due to its limited depth of acoustic penetration preventing accurate staging of locally advanced tumours (Garcia-Aguilar *et al*, 2002). In addition, specific analyses of tumours that have undergone a decrease in T stage post-RCT showed that up to 41% of these may harbour residual tumour deposits in the lymph nodes (Medich *et al*, 2001; Zmora *et al*, 2004).

We therefore feel that the conclusion of this study, based principally on the results of two papers, is a little premature. What does seem apparent, however, is that IHC and ssCP analysis of p53 yield unreliable and heterogeneous results. While DGS may hold promise, the question of whether such an expensive and labour-intensive technique will become useful in the clinical setting still remains to be answered.
